# Correction: Development of *N*-alkylated benzimidazole based cubosome hydrogel for topical treatment of burns

**DOI:** 10.1039/d4ra90127d

**Published:** 2024-10-25

**Authors:** Maubashera Nawaz, Sofia Hayat, Umer Farooq, Muhammad Adnan Iqbal, Syed Haroon Khalid, Tan Wen Nee, Kooi Yeong Khaw, Rabia Munir, Muhammad Umar Ijaz

**Affiliations:** a Department of Chemistry, University of Agriculture Faisalabad 38040 Pakistan adnan.iqbal@uaf.edu.pk; b Department of Pharmaceutics, Government College University Faisalabad 38000 Pakistan; c Department of Pharmaceutical Technology, Faculty of Pharmacy, Universiti Teknologi Mara (UiTM) Puncak Alam 42300 Selangor Malaysia; d Chemistry Section, School of Distance Education, Universiti Sains Malaysia 11800 Malaysia; e School of Pharmacy, Monash University Malaysia Jalan Lagoon Selatan Bandar Sunway 47500 Selangor Malaysia; f Department of Zoology, Wildlife and Fisheries, University of Agriculture Faisalabad 38040 Pakistan

## Abstract

Correction for ‘Development of *N*-alkylated benzimidazole based cubosome hydrogel for topical treatment of burns’ by Maubashera Nawaz *et al.*, *RSC Adv.*, 2024, **14**, 32008–32020, https://doi.org/10.1039/D4RA04816D.

The authors regret that there was an error in the sentence on lines 8–9 in the right column on page 32010 of the original article at the end of the “High resolution transmission electron microscope (HR-TEM)-morphology study” section. The text originally read ‘The process was conducted at the School of Distance Education, Universiti Sains, Malaysia’. This sentence should read ‘The process was conducted at Monash University Malaysia’.

The Royal Society of Chemistry apologises for these errors and any consequent inconvenience to authors and readers.

